# Supplementary material for: Human iPSC-Derived 2D and 3D Platforms for Rapidly Assessing Developmental, Functional, and Terminal Toxicities in Neural Cells
Source: Int J Mol Sci. 2021 Feb 14;22(4):1908. doi: 10.3390/ijms22041908 (PMC7918576; doi:10.3390/ijms22041908)
Supplement: Supplementary file 1 [file ijms-22-01908-s001.zip › Supplemental Materials/Supplemental Figures with legends.docx]

**Supplemental Figures**

**
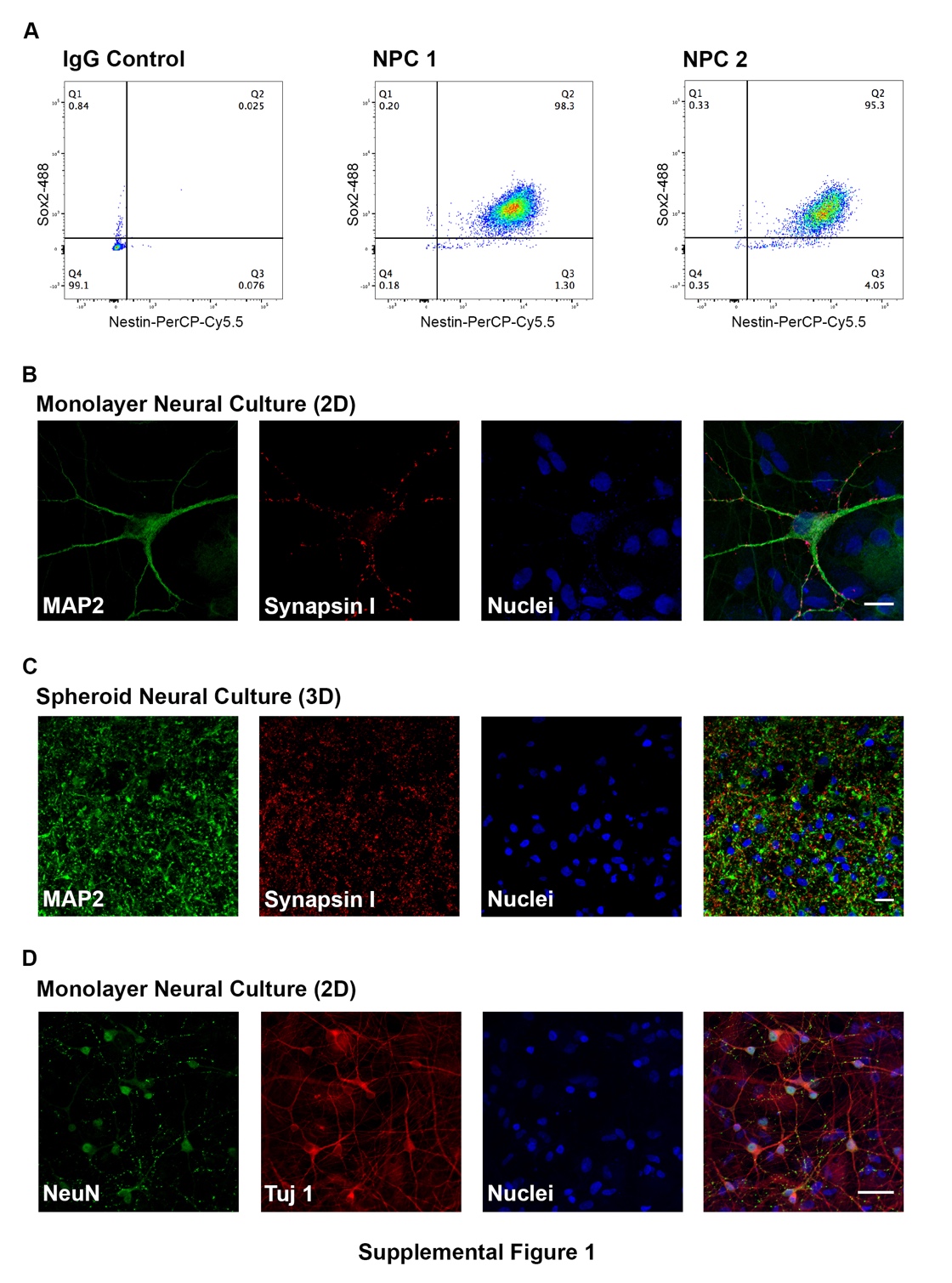
**

**Supplemental Figure 1 –** Characterization of hiPSC-derived neural cultures. (A) Flow analysis of the two NPC clones used in this study showing co-expression of Sox2 and Nestin in derived progenitor cells. (B-C) Immunocytochemistry (ICC) of 8-week-old 2D (B) and 3D (C) cultures showing the presence of maturity marker Synapsin I (Scale bar = 20 μm). (D) ICC of 8-week-old 2D neural cultures showing the presence of Tuj1 and NeuN (Scale bar = 50 μm).

**
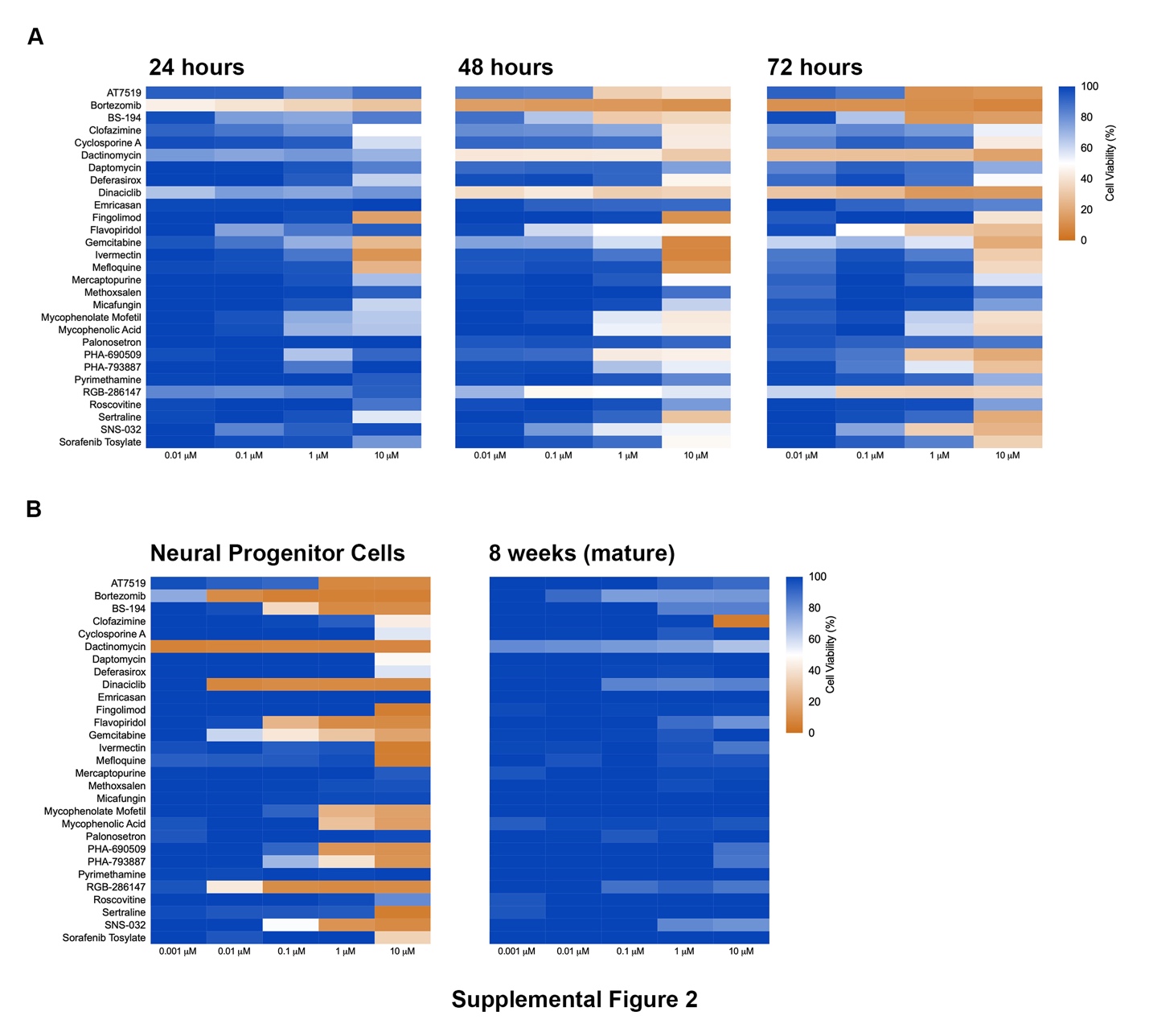
Supplemental Figure 2 –** Toxicity profile as a function of compound exposure time. (A) Heat map to determine the optimal compound exposure time. NPCs were exposed to compounds for 24, 48, or 72 hours, and viability was determined using CellTiter-Glo. The exposure time of 72 hours was chosen for all further experiments in this study. (B) Toxicity confirmation using a different hiPSC clone. Human iPSC-derived neural cultures from a second clone at progenitor (NPC) and mature (8-week-old) stages were exposed to compounds for 72 hours, and viability was determined using PrestoBlue. Graphs show heat map for cell viability compared to control (DMSO treated).


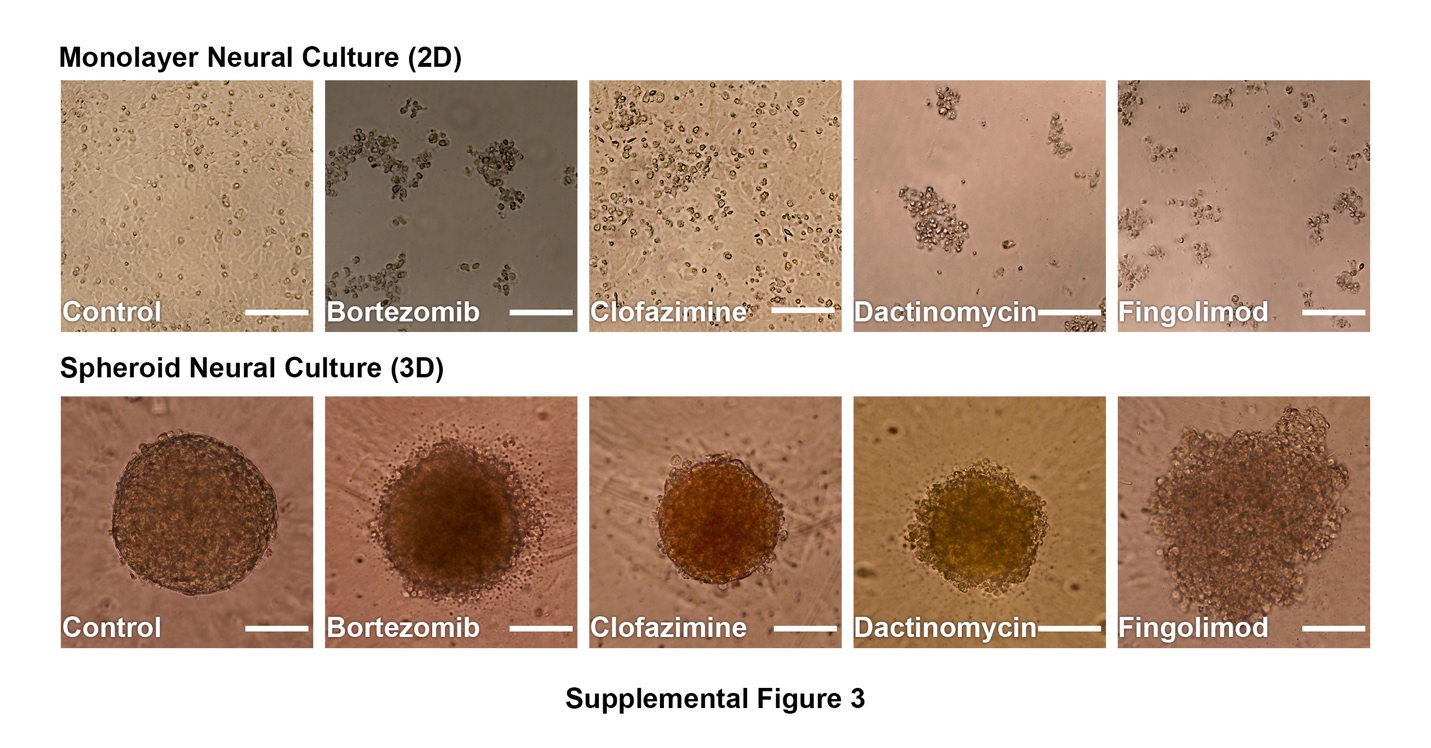


**Supplemental Figure 3 –** Brightfield microscopy images of progenitor cells after compound exposure. Representative images of toxic and non-toxic compounds after 48 hours exposure at 10 μM concentration. Monolayer (2D) and spheroid (3D) cultures are shown. Scale bar is 100 μm.

**
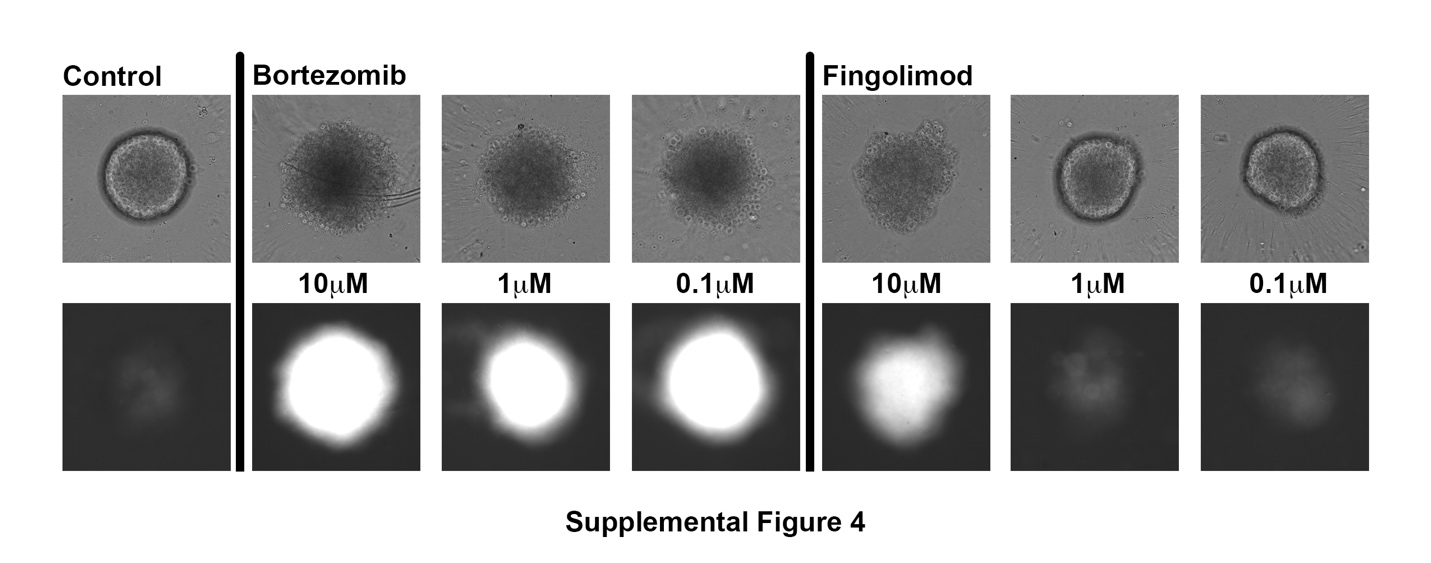
**

**Supplemental Figure 4 –** Brightfield and fluorescence imaging of spheroids (3D) from the Caspase-3/7 activation assay. Three compound concentrations are shown for two representative compounds. Images taken 48 hours after compound exposure. Fluorescence imaging used to quantify Caspase-3/7 activation relative to control (CellEvent^TM^ reagent).

**
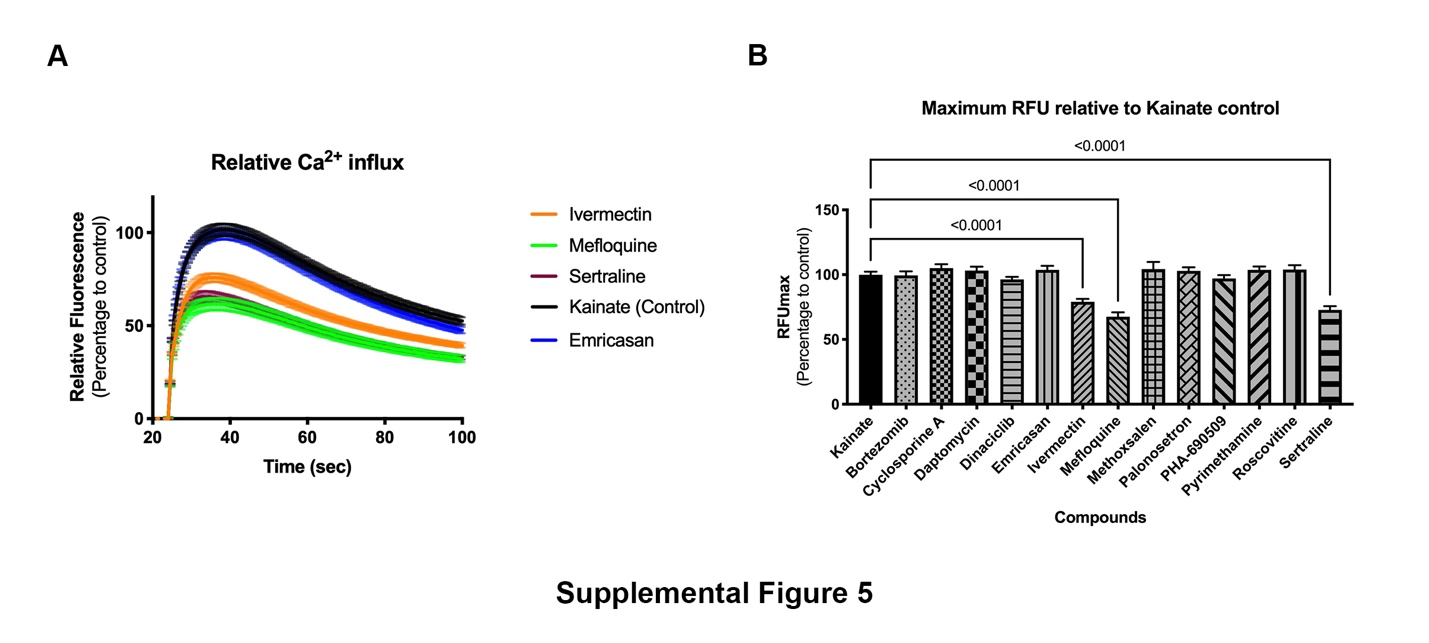
**

**Supplemental Figure 5 –** Functional response of 2D neural cultures to kainate stimulus. Graphs show relative fluorescence response over time (A) and maximum relative fluorescence (B) of kainate induced activity. Eight-week-old 2D neural cultures were exposed with selected compounds for 30 minutes before stimulus with kainate and functional response was recorded using FLIPR^TETRA^. Standard deviation is shown in both graphs. Number of replicates per compound = 14.
