# Supplementary figures and images for: Human iPSC-Derived 2D and 3D Platforms for Rapidly Assessing Developmental, Functional, and Terminal Toxicities in Neural Cells
Source: Int J Mol Sci. 2021 Feb 14;22(4):1908. doi: 10.3390/ijms22041908 (PMC7918576; doi:10.3390/ijms22041908)

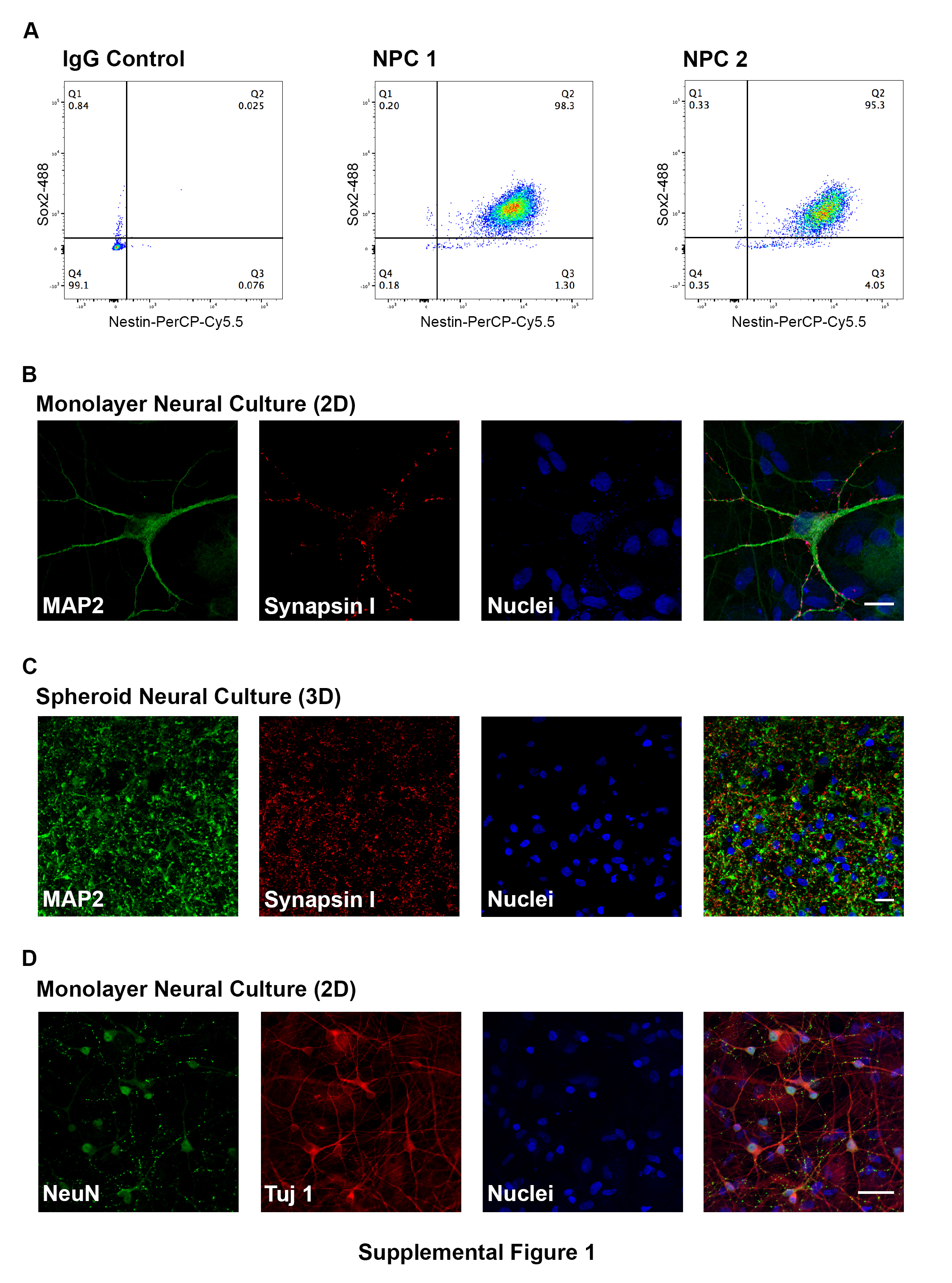

Supplement: Supplementary file 1 [file ijms-22-01908-s001.zip › Supplemental Materials/Supplemental Figure 1.jpg]

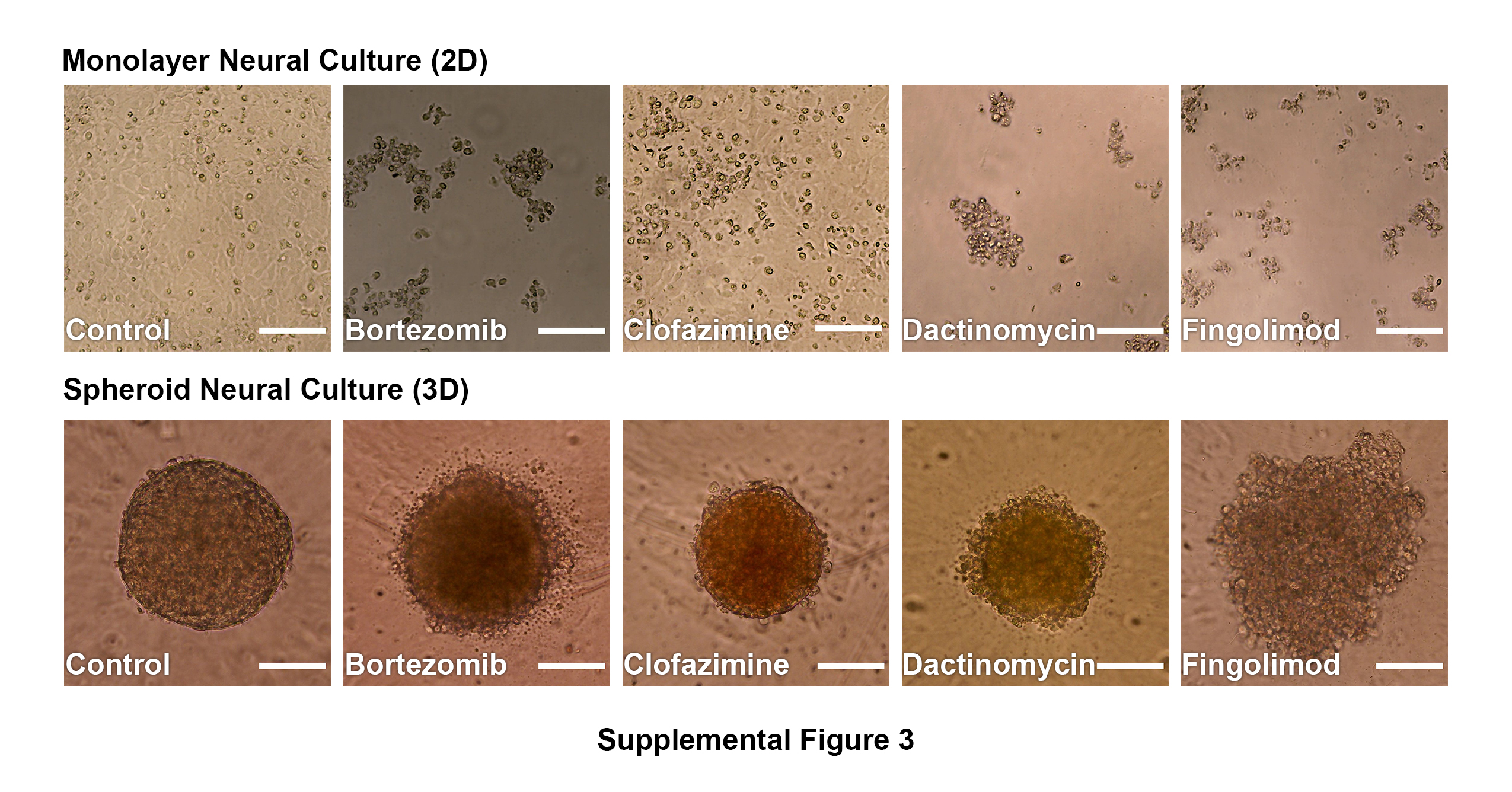

Supplement: Supplementary file 1 [file ijms-22-01908-s001.zip › Supplemental Materials/Supplemental Figure 3.jpg]

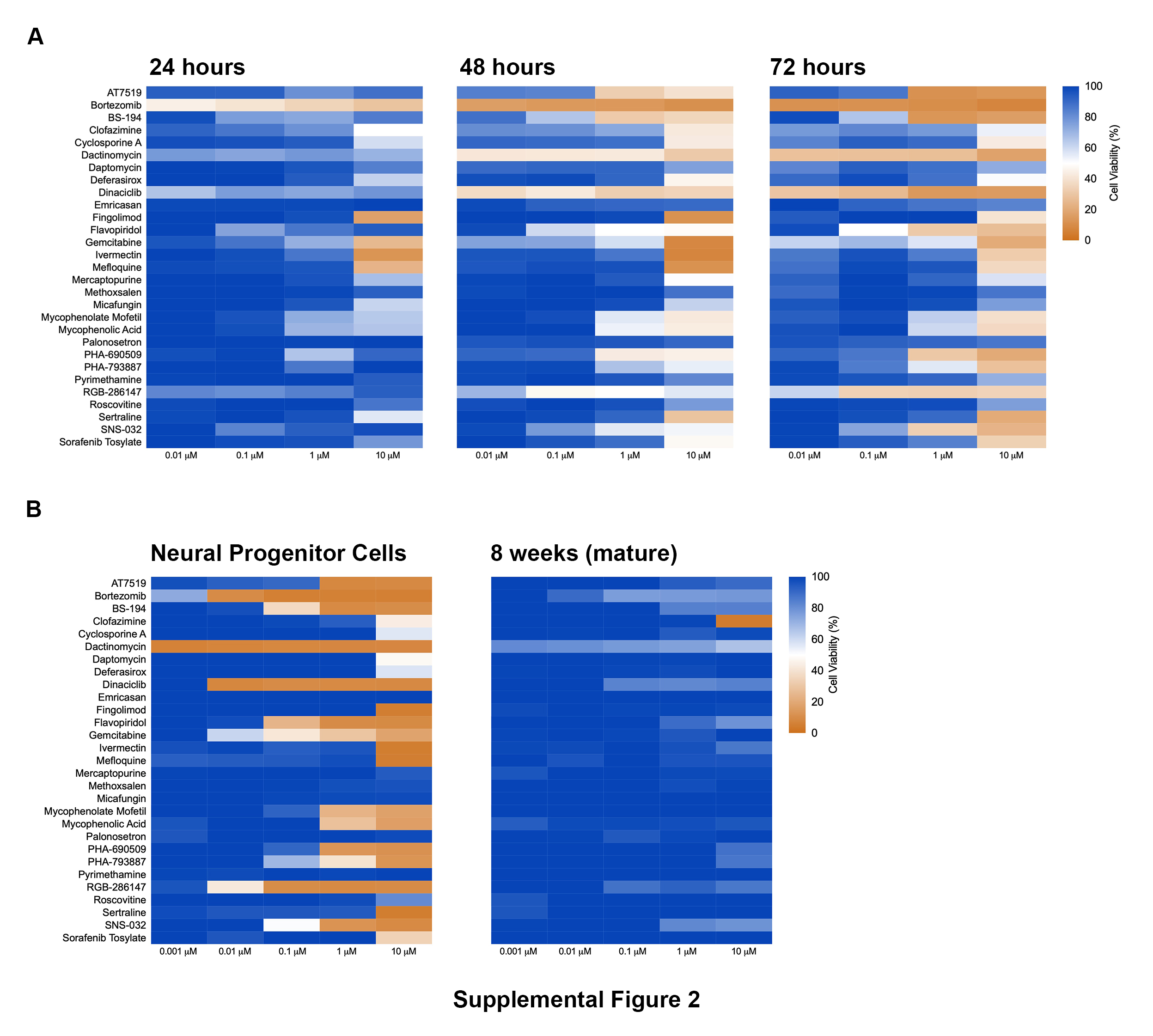

Supplement: Supplementary file 1 [file ijms-22-01908-s001.zip › Supplemental Materials/Supplemental Figure 2.jpg]

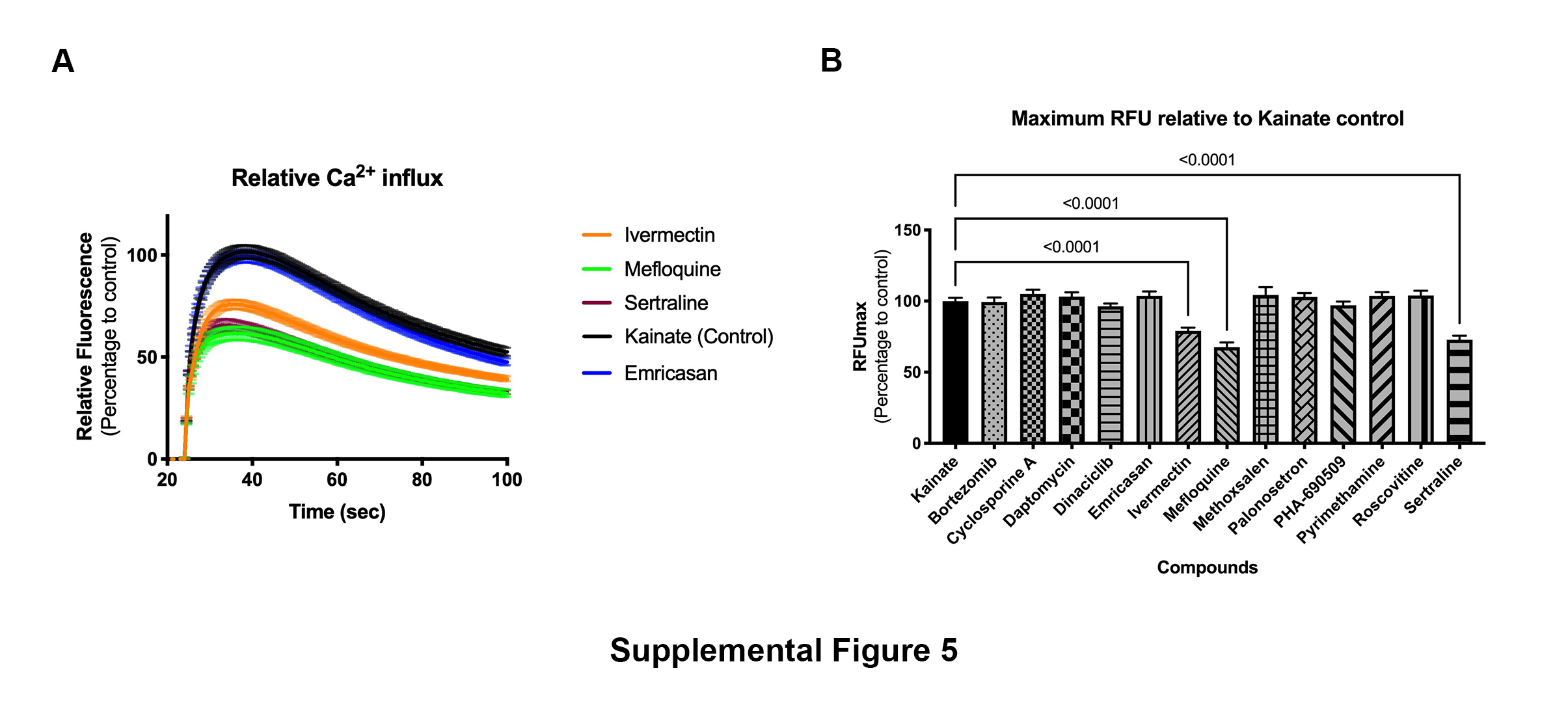

Supplement: Supplementary file 1 [file ijms-22-01908-s001.zip › Supplemental Materials/Supplemental Figure 5.jpg]

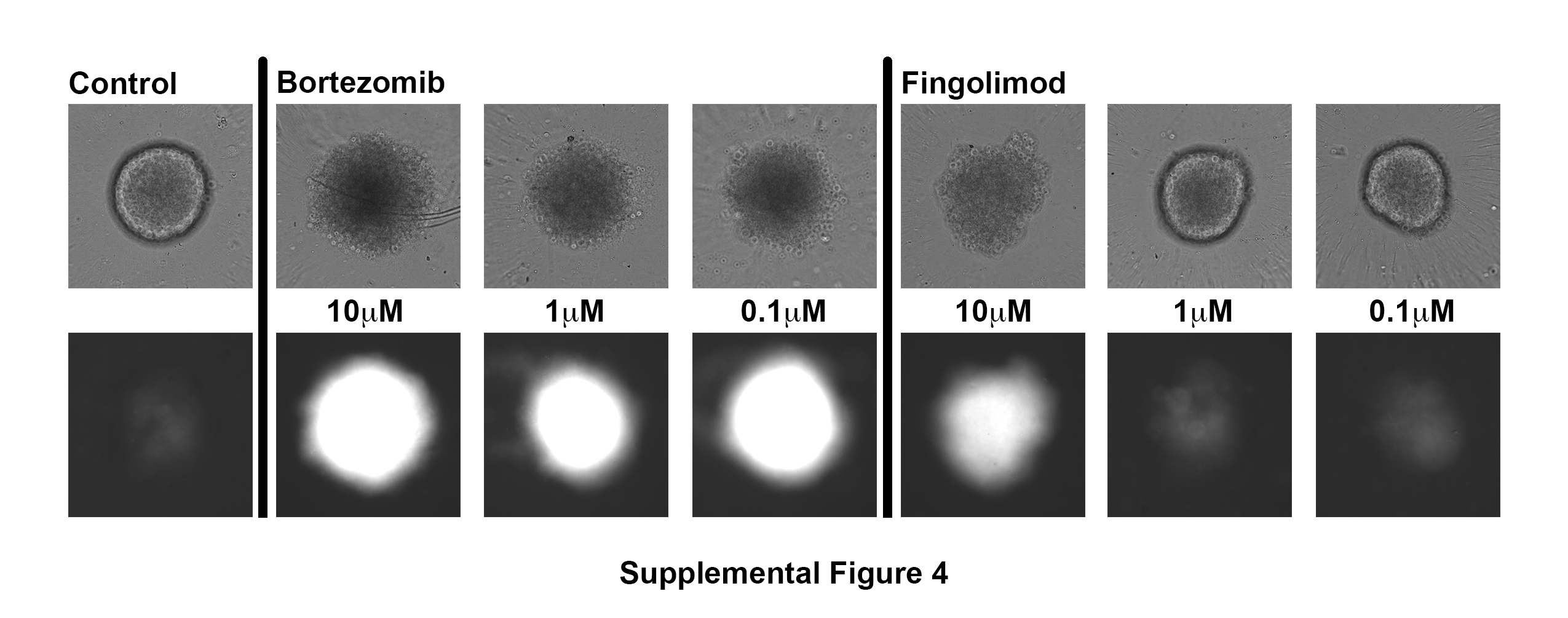

Supplement: Supplementary file 1 [file ijms-22-01908-s001.zip › Supplemental Materials/Supplemental Figure 4.jpg]
